# The Effects of an Increased Workload in Cataract Surgery Since the Pandemic in a Tertiary Care Clinic

**DOI:** 10.22336/rjo.2023.57

**Published:** 2023

**Authors:** Saadet Gültekin Irgat, Alpaslan Koç, Emine Çakar, Fatih Özcura

**Affiliations:** * Department of Ophthalmology, Kutahya Health Sciences University School of Medicine, Kutahya, Turkey

**Keywords:** cataract surgery, COVID-19, ophthalmology training, posterior capsule rupture

## Abstract

**Objective:** To evaluate the progress of cataract surgery in a training institution providing tertiary care since the removal of pandemic restrictions. To evaluate ocular and demographic characteristics of cataract cases in a tertiary care teaching institution since the lifting of pandemic restrictions.

**Methods:** Patients who underwent cataract surgery in our clinic in the pre-pandemic period September 2019-March 2020 (group 1, n=353) and in the post-pandemic period September 2021-March 2022 (group 2, n=459) were retrospectively screened. The cases were operated by two educator ophthalmologists and residents. The main parameters evaluated were cataract morphology, surgical parameters, and posterior capsular rupture complications.

**Results:** The case rate increased by 30% in group 2 once pandemic restrictions were relaxed. Preoperative best corrected visual acuity (BCVA) in groups 1 and 2 was 0.840±0.63 and 1.26±0.75 log MAR, respectively (p<0.001). The percentage of mature cataracts was 15.3% in group 1 and 31.2% in group 2 (p<0.001). Significantly higher cumulative dissipated energy (CDE), total aspiration time, and fluid amount (p<0.001 for all) were found in group 2.

During the training phase, 25.2% of the cases in group 1 and 24.6% in group 2 were performed by resident doctors (p=0.870). Residents in groups 1 and 2 had mature cataract case rates of 6.7% and 13.3%, respectively (p<0.001). The incidences of posterior capsule rupture in the instances of residents was 3.4% in group 1 and 4.4% in group 2 (p=0.498). A negative correlation (r=-0.424, p<0.001) between CDE and BCVA and a positive correlation (r=0.40, p<0.001) between cataract hardness and CDE were both found.

**Conclusions:** The number of cataract surgeries increased after the COVID-19 pandemic. Poor vision and increasing rates of mature cataracts are other effects of this backlog. Residents have to deal with challenging cases. Our results are just the tip of the iceberg. Urgent planning is needed to deal with the remaining cases.

**Abbreviations:** COVID-19 = coronavirus infection, PCR = posterior capsular rupture, BCVA = best corrected visual acuity, IOP = intraocular pressure, CDE = cumulative dissipated energy, TAT = total aspiration time, ZD = zonular separation

## Introduction

A new coronavirus infection (COVID-19) emerged in Wuhan, China, in December 2019 [**1**]. The World Health Organization called COVID-19 a pandemic on March 11, 2020, because it spread so quickly and posed a threat to public health around the world [**2**]. To prevent the spread of the disease, some governments have implemented isolation measures.

The 16th of March, 2020, marked the start of isolation precautions in Turkey. The COVID-19 pandemic has created a significant challenge in the provision of surgical care globally, as elective operations and outpatient appointments have been postponed [**3**]. Being a branch of medicine that deals with elective surgeries, Ophthalmology has been significantly affected. During the first wave of COVID-19, global eye surgeries, including cataract surgery, decreased by 90% in the US, Europe, and India [**4**-**6**]. Cataract surgeries have been suspended for 3 months. Whereas the pandemic period was not only limited to 3 months.

Increasing cases and restrictions have caused postponing the weekly surgical case lists by affecting the delivery of healthcare services. Furthermore, the delay in hospital admissions of patients who suffered from cataracts, because of the precautions and the worry of getting infected of the patients’ has created a massive patient burden [**7**]. Not only did the number of operations rise, but the cataracts of the patients who were delayed and awaited advanced, and a rise in the prevalence of mature cataracts were noted [**8**,**9**]. Along with this, the rate of posterior capsular rupture (PCR), which went up when elective surgery was put on hold, was linked to a drop in technical skills, especially fine motor skills [**10**,**11**].

At the same time, COVID-19 infection also affected the academic and professional development of surgery residents, causing significant disruptions in all kinds of medical education and training [**12**-**15**].

This study aims to evaluate the outcomes of the COVID-19 pandemic on cataract surgery two years later in a tertiary university hospital in Turkey.

## Materials and methods

The data of 812 patients who underwent phacoemulsification surgery for cataracts in our ophthalmology clinic between September 2019 and March 2020 (group 1, n=353) and September 2021-March 2022 (group 2, n=459) were retrospectively analyzed. Informed consent was obtained from the patients. The procedures used in this study adhered to the tenets of the Declaration of Helsinki, and they were approved by the local ethics committee. In addition to demographic data, best corrected visual acuity (BCVA, logMAR) before and after surgery, intraocular pressure (IOP), cataract classification - cataract type (a) immature-subcapsular, nuclear, cortical, and mix (b) mature-white, intumescent, black-nigra cataract, and pupil characteristics - small pupil, pseudoexfoliation, were recorded. Surgery-related phacoemulsification parameters (cumulative dissipated energy - CDE, total aspiration time - TAT, fluid amount - ml) and PCR, zonular separation (ZD), were screened.

*Statistical analysis*

SPSS (v. 22.0 for Windows, SPSS INC, Chicago, IL) was used for statistical analysis. After checking for normality with the Kolmogorov-Smirnov test, both parametric and non-parametric methods were applied, respectively. Categorical variables were compared using Fisher’s exact probability test and chi-square test, while Student’s t-test and Mann-Whitney test were used to compare continuous variables. Categorical data were defined as ratios, and continuous data as mean ± standard error. Statistical significance was considered as a p-value less than 0.05.

## Results

The patients in the study had a mean age of 67.09±9.6 years, and 56% of them were male.

No difference between the two groups in terms of age, gender, laterality, or IOP distribution (**Table 1**) was observed. Demographic and ocular parameters did not differ amongst surgeons (p>0.05 for all). A 30% rise in the number of patients who received surgery was observed in group 2.

**Table 1:** Ocular and demographic characteristics in study groups

variable	Group 1	Group 2	p
Age at surgery (years), mean ± SE	67.02±9.39	67.15±9.90	0.84
male/female/(% male)	197/156 (55.8%)	261/198 (56.9%)	0.77
BCVA (logMAR)	0.84±0.63	1.26±0.75 logMAR	<0.001
Eye (Right/left)	182/171	268/191	0.055
Mature/Brown/Blackcataract (%)	54 (15.3%)	143 (31.2%)	<0.001
CDE	7.48±6.46	10.15±7.43	<0.001
TAT(sn)	5.6±2.04	6.14±2.19	<0.001
Irrigation fluid (ml)	81.31±24.98	88.72±29.36	<0.001
Small pupil (%)	19 (5.4%)	28 (6.1%)	0.06
PEX	28 (7.9%)	37 (8.1%)	0.068
Capsule ring	7 (2.0%)	11 (2.4%)	0.812
PCR	6 (1.7%)	11 (2.4%)	0.332
CDE = cumulative dissipated energy, TAT = total aspiration time, PCR = posterior capsule rupture, PEX = pseudoexfoliation, BCVA = best corrected visual acuity			

Preoperative BCVA in groups 1 and 2 was 0.840±0.63 logMAR and 1.26±0.75 logMAR, respectively (p<0.001). According to the cataract classification, the percentage of mature, brown-black cataracts was 15.3% in group 1 and 31.2% in group 2 (p<0.001). Significantly higher CDE, TAT, and fluid levels (p<0.001 for all) were observed in group 2. In group 1, 5.4% of the cases had small pupils and 7.9% had pseudoexfoliation. A capsule tension ring had to be implanted in 2.0% of cases due to zonular dialysis (p=0.812). In group 2, these rates were 6.1%, 8.1%, and 2.4%, respectively (all p>0.05). In group 1, posterior capsule rupture complications were 1.7%; in group 2, they were 2.4% (p=0.332).

In groups 1 and 2, respectively, educator ophthalmologists were in charge of 74.8% and 75.4% of the cases (p=0.870). The rates of nuclear cataracts were 33.0% in group 1 and 21.4% in group 2, while the rates of mature cataracts increased from 18.2% to 37.0%, (p<0.001). The PCR complication rate was not statistically significant between the two groups (p=0.0399) (**Table 2**).

**Table 2:** Clinical characteristics of cases examined by residents and educators in groups 1 and 2

variable	Group 1	Group 2	p
Surgeon (educator ophthalmologist)			
number of cataract surgeries (n,%)	264 (74.8%)	346 (75.4%)	0.870
nuclear cataract rate	87 (33.0%)	74 (21.4%)	<0.001
mature cataract rate	48 (18.2%)	128 (37.0%)	<0.001
BCVA	0,90±0.67	1,38±0,76	<0.001
CDE	7.16±6.5	10.08±7.66	<0.001
TAT	5.40±1.89	5.73±2.01	0.043
Liquid	78.28±22.12	82.31±26.81	0.048
PCR	3 (1.1%)	6 (1.7%)	0.399
Ophthalmic residents			
number of cataract surgeries (n,%)	89 (25.2%)	113 (24.6%)	0.870
nuclear cataract rate	39 (43.8%)	29 (25.7%)	<0.001
mature cataract rate	6 (6.7%)	15 (13.3%)	<0.001
BCVA	0.67±0.47	0.89±0.60	0.006
CDE	8.44±6.26	10.36±6.71	0.039
TAT	6.19±2.37	7.40±2.27	<0.001
Liquid	90.29±30.39	108.38±28.19	<0.001
PCR	3 (3.4%)	8 (4.4%)	0.498
CDE = cumulative dissipated energy, TAT = total aspiration time, PCR = posterior capsule rupture, PEX = pseudoexfoliation, BCVA = best corrected visual acuity			

During the training phase, 25.2% of the cases in group 1 and 24.6% in group 2 were performed by resident doctors (p=0.870) (**Table 2**). The resident doctors’ cases had nuclear cataract levels, with rates of 43.8% in group 1 and 25.7% in group 2, respectively (p<0.001). Group 1 had 6.7% of mature cataracts, whereas group 2 had 13.3%(p< 0.001). In the cases of resident doctors, CDE was 8.44±6.26 in group 1 and 10.36±6.71 in group 2 (p=0.039). The frequency of PCR in the instances of residents was 3.4% in group 1 and 4.4% in group 2 (p=0.498). A negative correlation (r=-0.424, p<0.001) between CDE and BCVA (logMAR) and a positive correlation (r=0.40, p<0.001) between cataract hardness and CDE were both found. 

## Discussion

The World Health Organization declared COVID-19 a pandemic on March 11, 2020, due to its rapid spread and global threat to public health [**2**]. Most clinics stopped performing routine clinical and surgical procedures during the initial few months of the pandemic epidemic and limited their operations to treating emergencies alone [**16**-**18**].

A cataract is the world’s second most common cause of preventable blindness [**20**]. In most cases, cataract surgery is an elective procedure. Patients and their loved ones were worried about the possibility of contracting COVID-19 in the course of cataract surgery during the pandemic period [**20**,**21**]. However, it is well-recognized that delaying cataract surgery can cause decreased vision, which can lead to car accidents, home mishaps, and depression [**22**-**26**].

The suspension of elective cataract surgeries during the COVID-19 surge had a significant and long-term impact on ophthalmology, resulting in a large patient burden over the coming months and years [**6**,**7**]. Even in the most optimistic picture, they predicted that it would take 9 months to overcome the workload [**7**]. According to a study conducted in India, it was found that after the restrictions were lifted, the number of cases increased compared to the period before the pandemic [**6**]. According to our findings, the number of cataract cases increased by 30% after the restrictions were lifted, compared to the period before the pandemic. The addition of pending cataract cases to the system following the suspension of surgeries and restrictions during the pandemic appears to have resulted in a significant surgical workload.

We found that the mean LogMAR visual acuity was worse and the surgical measures CDE, TAT, and fluid volume were higher in patients administered after lockdowns. These results indicated that the COVID-19 pandemic delayed cataract procedures, resulting in the development of mature and hyper-mature cataracts. As a result, there were two main reasons why the prevalence of mature white cataracts increased during the pandemic: 1 - Longer patient wait times for cataract treatments, and 2 - Patients delaying visits to ophthalmologists out of fear of contracting COVID-19 infection.

As a result of longer lockdown periods during the epidemic, there are now more people with advanced cataracts, which is very concerning. This could lead to reduced visual acuity and more surgical problems. Poor visual acuity has been linked in studies to depression, home mishaps, and auto accidents [**22**-**26**]. This entails an increase in workload across several industries.

In addition, many eye surgeons stopped performing microsurgery for several months during the pandemic and were assigned to medical and intensive care units. It has been shown that interruption of regular surgical practice caused regression in technical skills [**10**].

It seemed inevitable that advanced cataracts and a short break from the surgery increased surgical complications. Therefore, we looked into PCR rates in our study as one of the risks associated with cataract surgery.

We found that the mean LogMAR visual acuity was worse and the surgical measures CDE, TAT, and fluid volume were higher in patients admitted after lockdowns. These results indicated that the COVID-19 pandemic delayed cataract procedures, resulting in the development of mature and hyper-mature cataracts. In this regard, baseline BCVA in our study measured 0.84±0.63 logMAR in group 2 and 1.26±0.75 logMAR in group 1 (p<0.001). In our cases, the prevalence of developed mature cataracts increased from 15.3% to 31.2%. In an Indian study, BCVA dropped from 1.39±1.05 LogMAR after restrictions to 1.51±1.08 LogMAR (P=0.001), and the prevalence of mature cataracts rose from 14.9% to 19.07% [**27**]. As a result, there were two main reasons why the prevalence of mature white cataracts increased during the pandemic: 1 - Longer patient wait times for cataract treatments, and 2 - Patients delaying visits to ophthalmologists out of fear of contracting COVID-19 infection.

It has been shown that technical skills deteriorate when routine surgical practice is stopped for any reason [**10**]. During the pandemic, a lot of eye surgeons stopped performing microsurgery for a while and were placed in medical and intensive care units. In advanced cataracts and the case of a short break from the operation, the complications of surgery were expected to increase. Therefore, we investigated PCR rates in our study as one of the risks associated with cataract surgery.

In our study, PCR was 1.7% in group 1 and 2.4% in group 2 (p=0.332). According to the study of Theodoraki et al., after a surgical curtailment of 9 months, the PCR rate increased from 1.67% to 3.55% and it was higher than it had been during the pre-pandemic period [**10**]. In consonance with another study, following the pandemic, the PCR frequency reached from 0.99% to 1.62% [**11**]. In agreement with the investigators, surgical activity limitations during lockdowns increased the risk of PCR [**10**,**11**]. However, even though the PCR complication rate rose in our instances, this was not statistically significant. The low PCR rates can be explained by continuing surgery while treating advanced cataract cases as emergencies in our clinic during the pandemic period, as well as by the skill of the surgeons. The fact that some studies were conducted right away after surgical restraints were lifted explained their high PCR rates.

The residency years are crucial for developing surgical techniques and concentrating one’s focus. The COVID-19 era was a very unfortunate time for surgical assistants who were just starting their professions. In many hospitals, residents have been at the forefront of COVID-19 services. When they resumed surgery, they had to contend with anxiety, tension, and a drop in technical training [**14**]. In almost all earlier studies, the pandemic has been proven to harm ophthalmology education [**13**,**27**-**30**].

In an Indian survey, nearly 80% of residents agreed that quarantine had negatively affected their surgical education [**27**]. Approximately half of the trainees polled in Portugal stated that they did no surgical procedures other than intravitreal injections [**13**]. A survey of ophthalmology residents in the United Kingdom evaluating the effect of COVID-19 on education revealed that the most frequently expressed concern was a lack of cataract surgery training [**29**,**30**]. Although there was no difference in the rates of resident cases between the two groups in our study, they faced difficult cases due to an increase in the incidence of mature cataracts, and the complication rates were acceptable. Although resident training was hampered in the early months of the pandemic, perhaps, overall, difficult cases were included in training and gained an advantage in learning to deal with difficult cases.

The limitations of our study were that it was single-centered and retrospective.

Our research demonstrated that after the pandemic, there was at least a 30% increase in the rates of cataract surgery, the procedure that ophthalmologists perform most frequently. Additionally, the surgical parameters showed a correlation with the rise in advanced cataract rates. The number of cases examined by ophthalmology residents did not change, but the kinds of cataracts they operated on did, and by handling more difficult cases, they learned new skills. There has been an increase in PCR complications, though it was not statistically significant. The pandemic was a natural occurrence that affected all societal groups and the entire world. Our results highlighted only the tip of the iceberg in cataract surgery. The increased workload, however, should have been managed in a planned manner after these disasters were over without endangering the public’s health, and research should have been done in this area.

## Conclusion

To avoid cataract-related blindness, proactive steps must be taken to overcome this backlog. The data obtained from our study may help plan health service delivery in similar situations in the future.

**Conflict of Interest**

The author(s) declared no potential conflicts of interest concerning the research, authorship, and/or publication of this article.

**Informed Consent and Human and Animal Rights Statements**

Every individual involved in this study has given their informed consent.

**Authorization for the use of human subjects**

Ethical approval: The clinical research ethics committee at Kutahya Health Sciences University, Turkey has approved the research about human use, which complies with all applicable national regulations, institutional policies, and the principles of the Helsinki Declaration (Date 6.04.2022/ no: 2022/04-13).

**Acknowledgments**

None.

**Sources of Funding**

The author(s) received no financial support for the research, authorship, and/or publication of this article.

**Disclosures**

None.
